# Supplementary material for: Proteolytic processing of the unique F1-ATPase subunit α in procyclic Trypanosoma brucei
Source: Sci Rep. 2026 Apr 28;16:27787. doi: 10.1038/s41598-026-49778-4 (PMC13545225; doi:10.1038/s41598-026-49778-4)
Supplement: Supplementary file 1 [file 41598_2026_49778_MOESM2_ESM.pdf]

Proteolytic Processing of the Unique F1-ATPase Subunit α in Procyclic *Trypanosoma brucei*

Full uncropped blots for
Figure 1B and 1D;
Figure 2 D, E, F,
Figure 3E, D,
Figure 4 B, D,
Figure 5B,
Figure 6B,
Figure S1 C, D,
Figure S2

Figure 1B

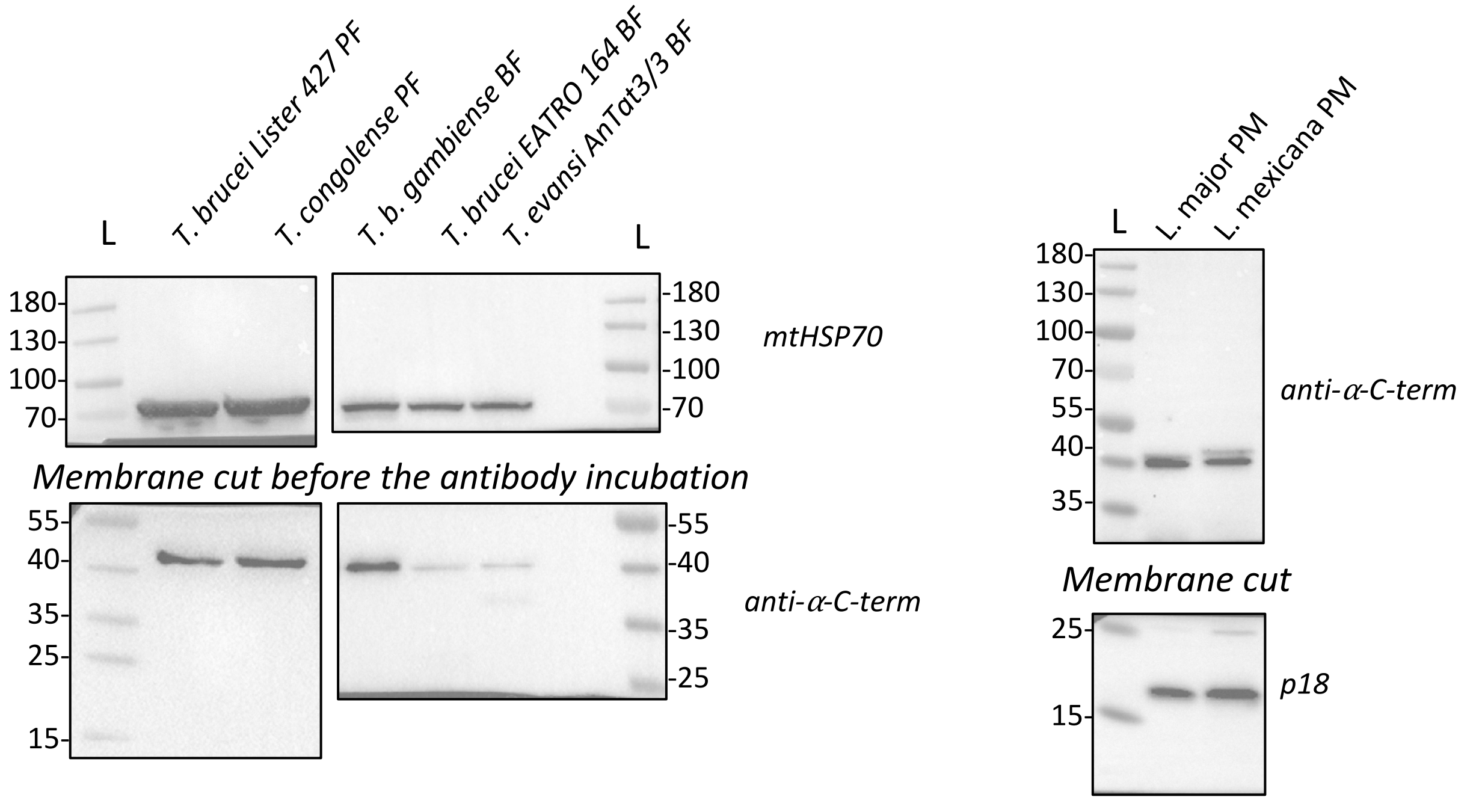

Figure 1D

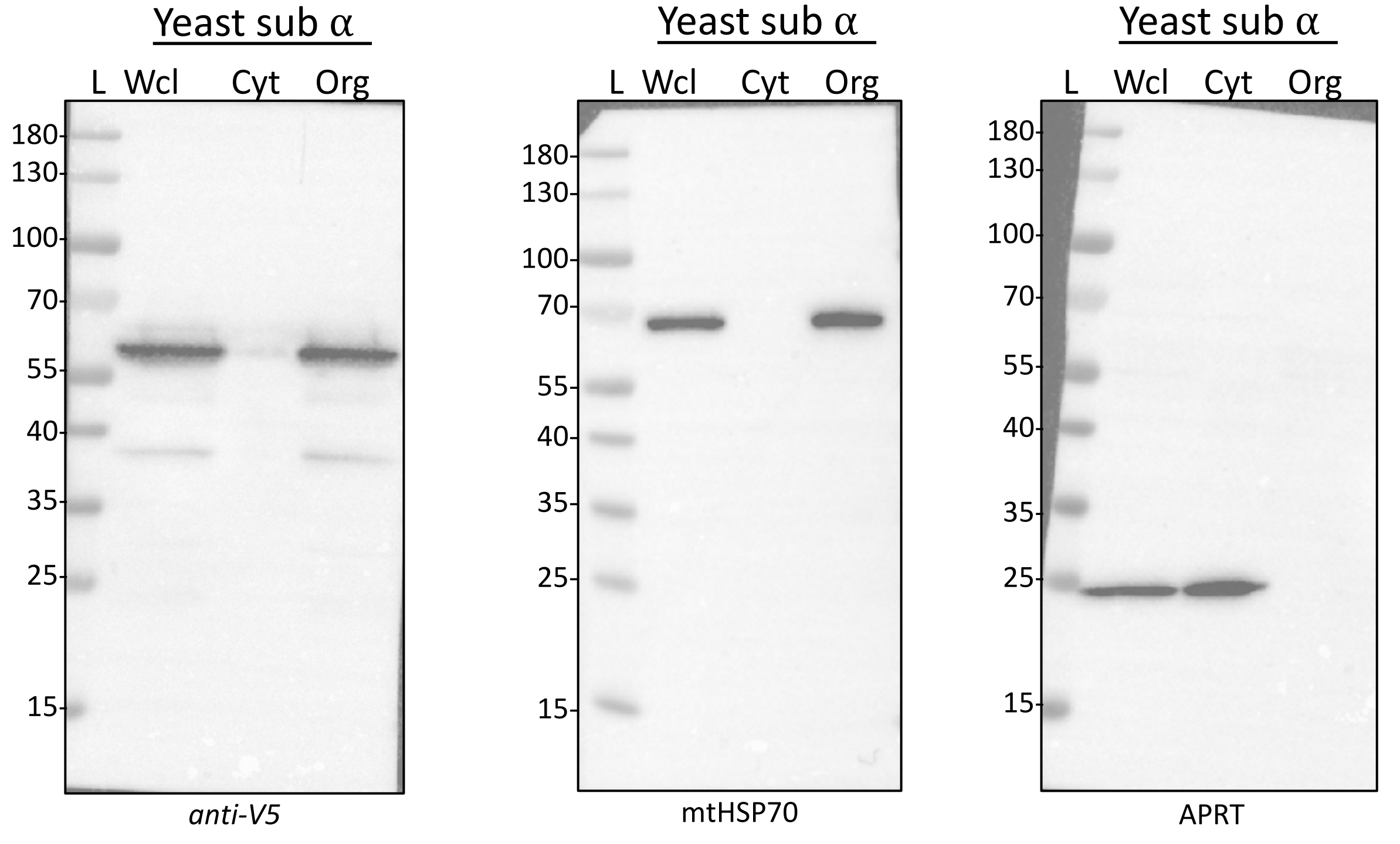

Figure 2D

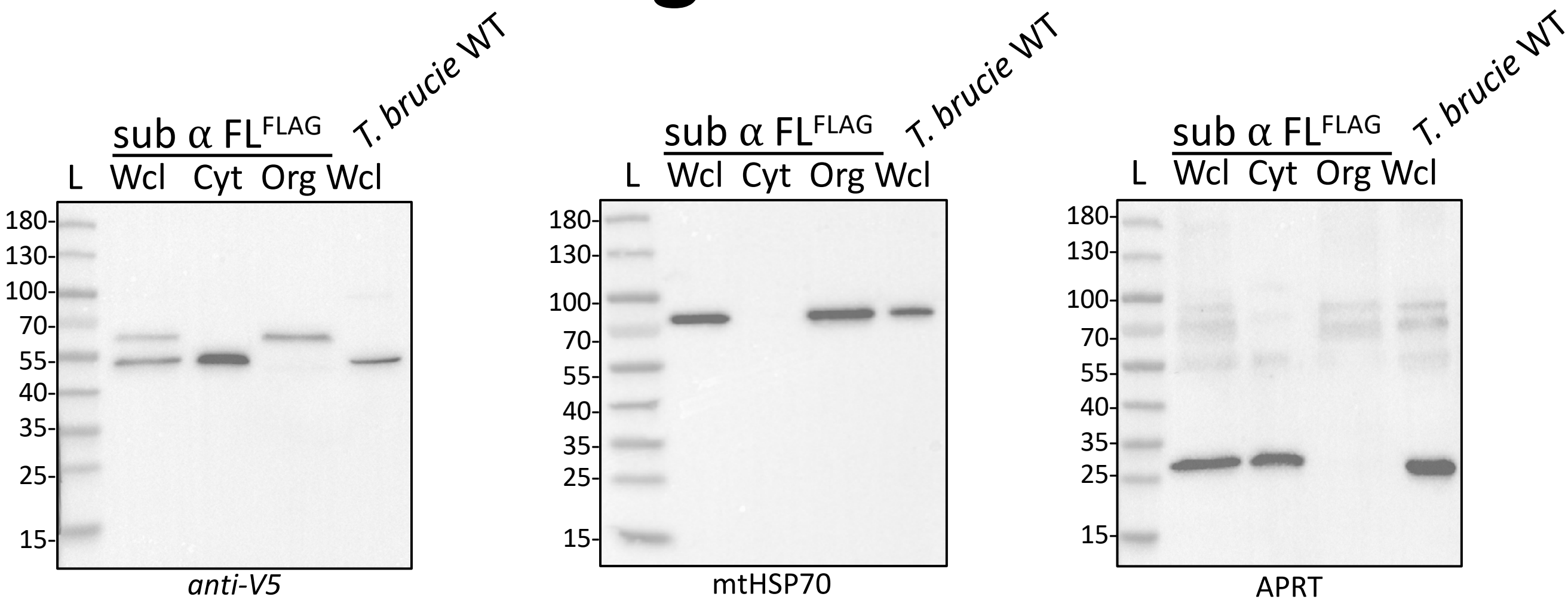

Figure 2E

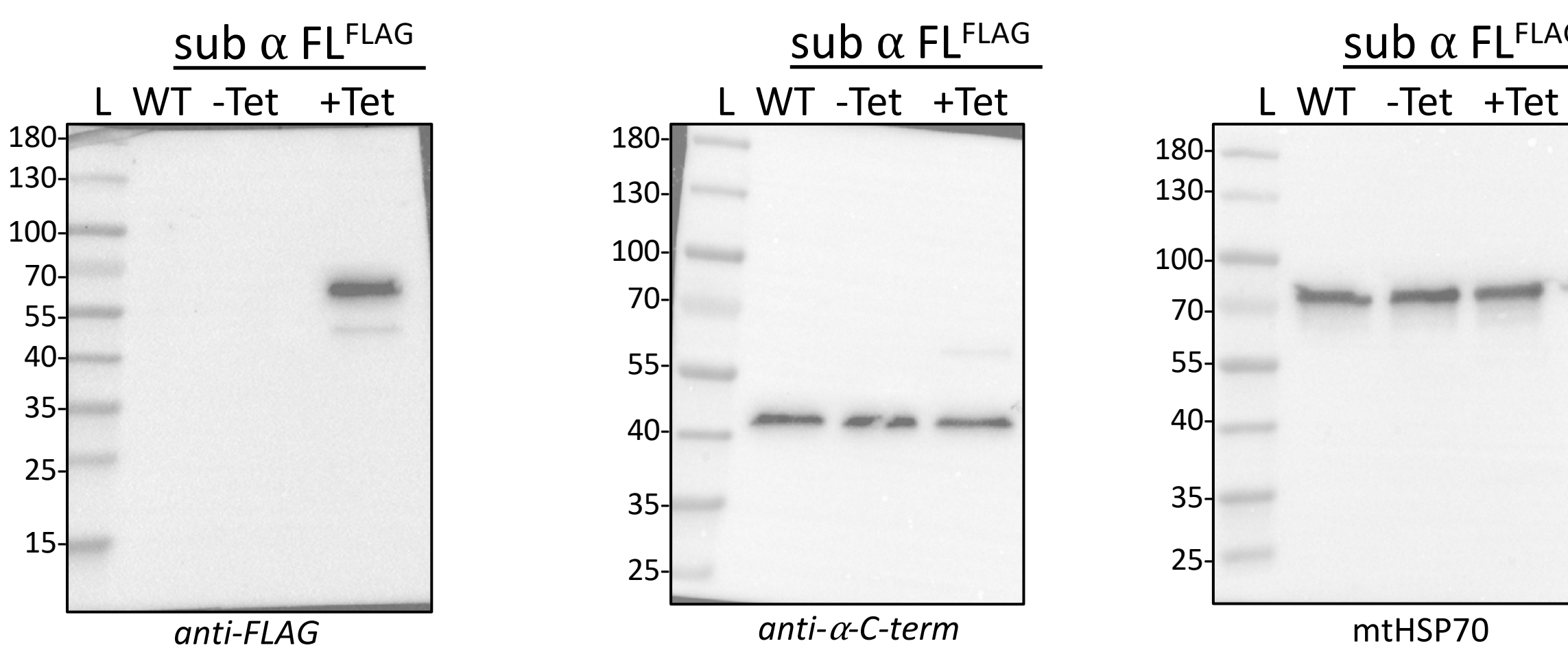

Figure 2F

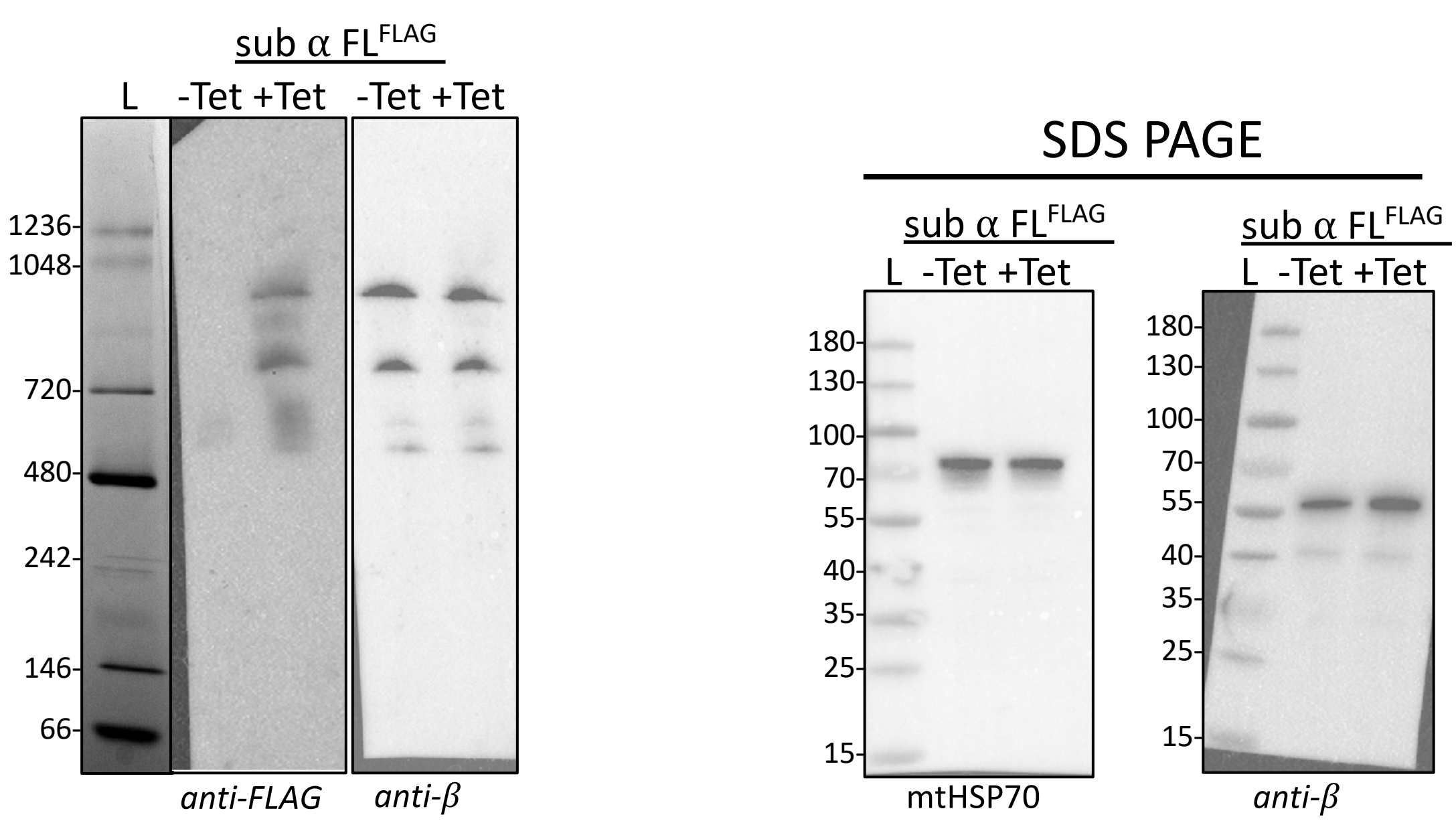

Figure 3D

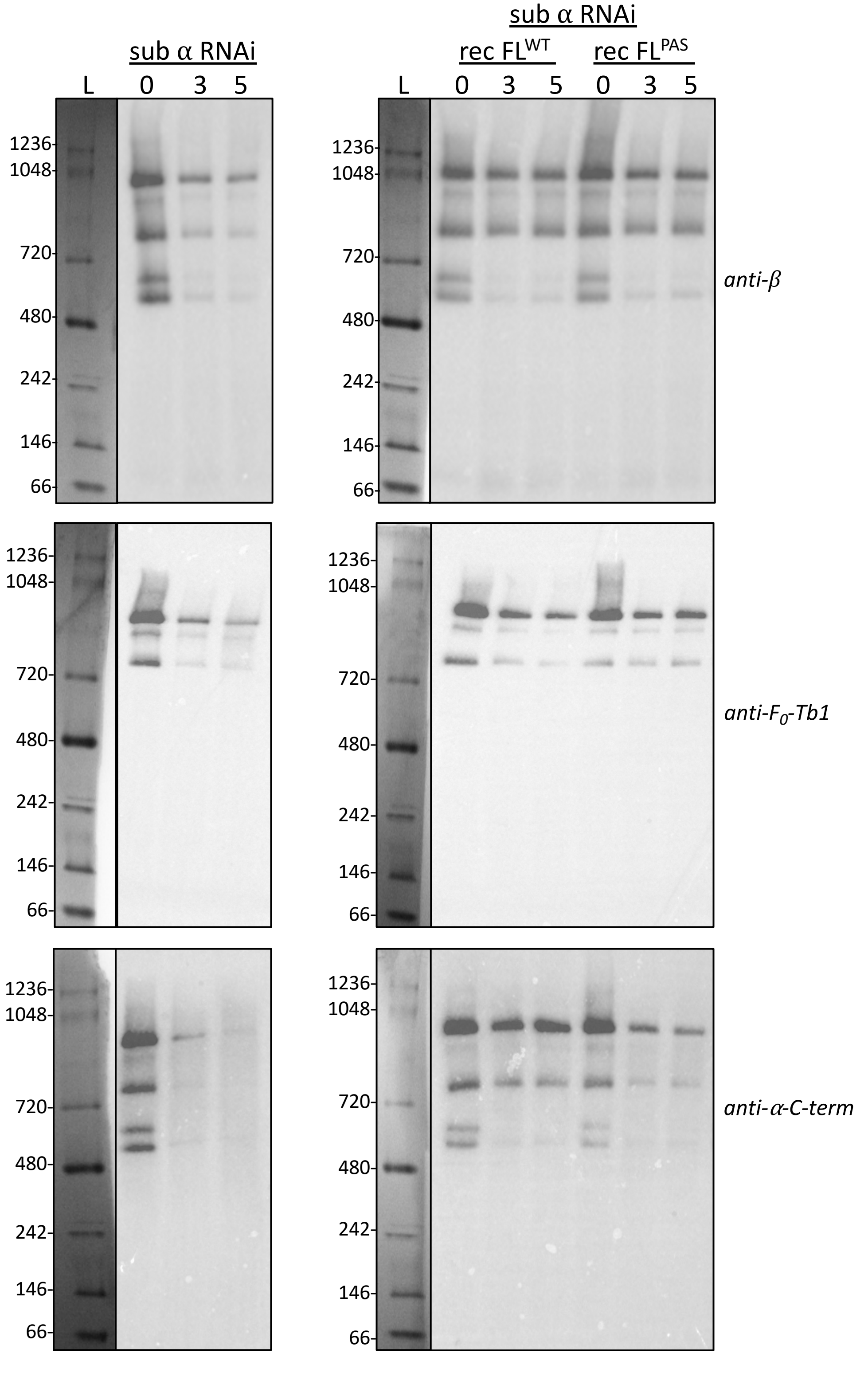
sub α RNAi
L 0 3 5
sub α RNAi
rec FL^WT rec FL^PAS
L 0 3 5 0 3 5
1236
1048
720
480
242
146
66
anti-β
anti-F0-Tb1
anti-α-C-term

Figure 3E

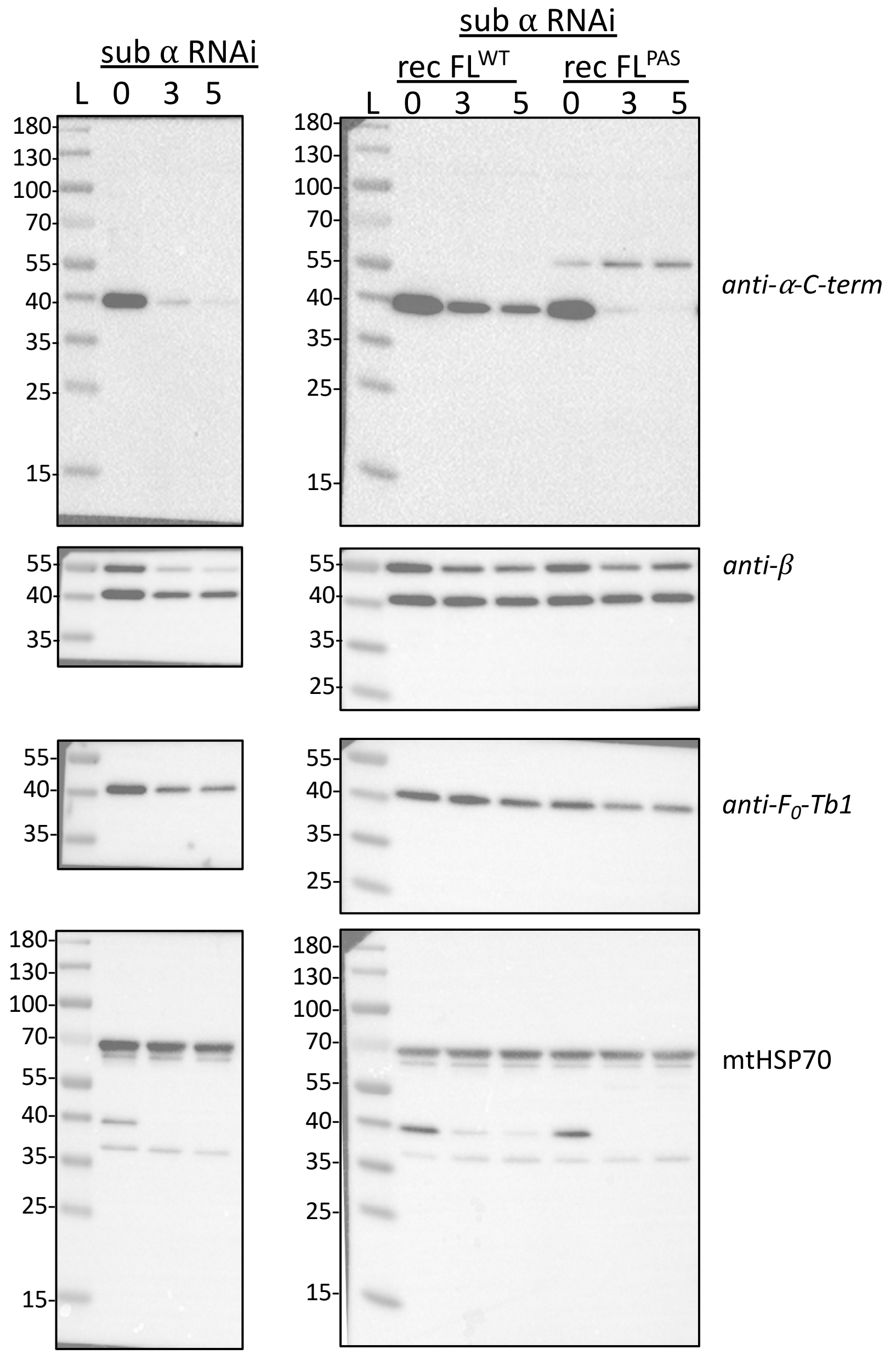
sub α RNAi
L 0 3 5
sub α RNAi
rec FL^WT
rec FL^PAS
L 0 3 5 0 3 5
180
130
100
70
55
40
35
25
15
anti-α-C-term
anti-β
anti-F0-Tb1
mtHSP70

Figure 4B

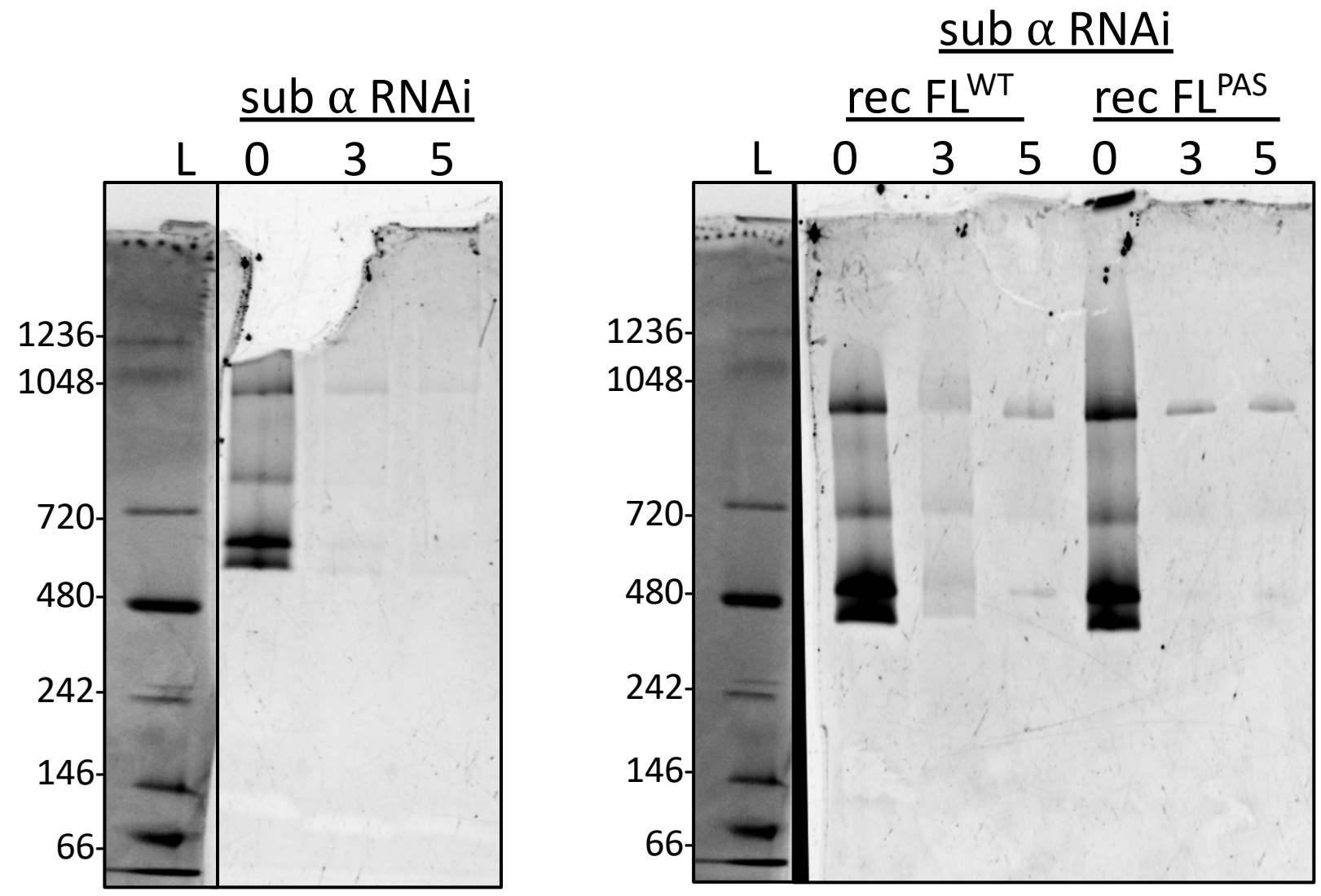

Figure 4D

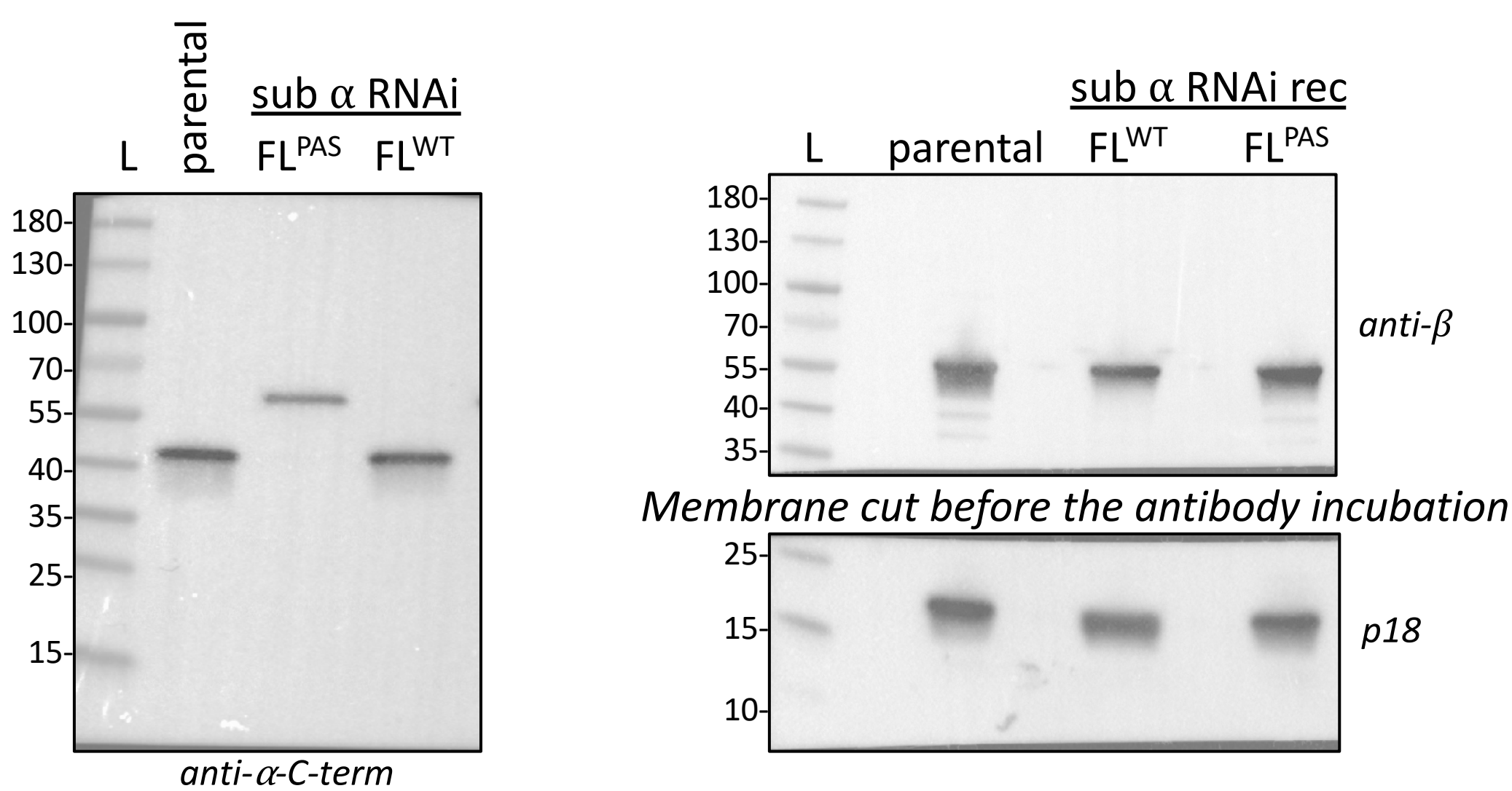

Figure 5B

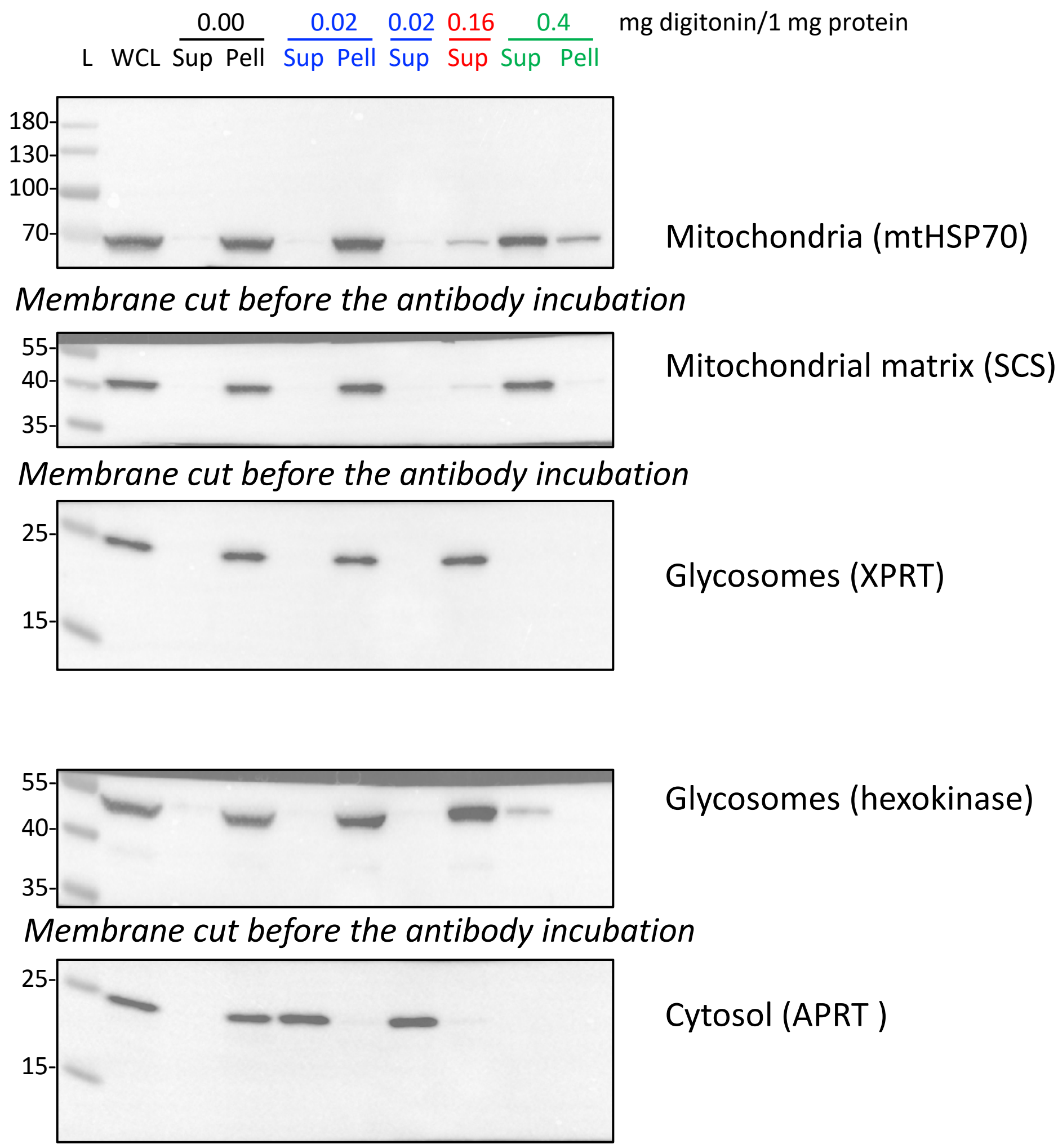
0.00
0.02
0.02
0.16
0.4
mg digitonin/1 mg protein
L
WCL
Sup
Pell
Sup
Pell
Sup
Sup
Sup
Pell
180
130
100
70
Mitochondria (mtHSP70)
Membrane cut before the antibody incubation
55
40
35
Mitochondrial matrix (SCS)
Membrane cut before the antibody incubation
25
15
Glycosomes (XPRT)
55
40
35
Glycosomes (hexokinase)
Membrane cut before the antibody incubation
25
15
Cytosol (APRT)

Figure 6B

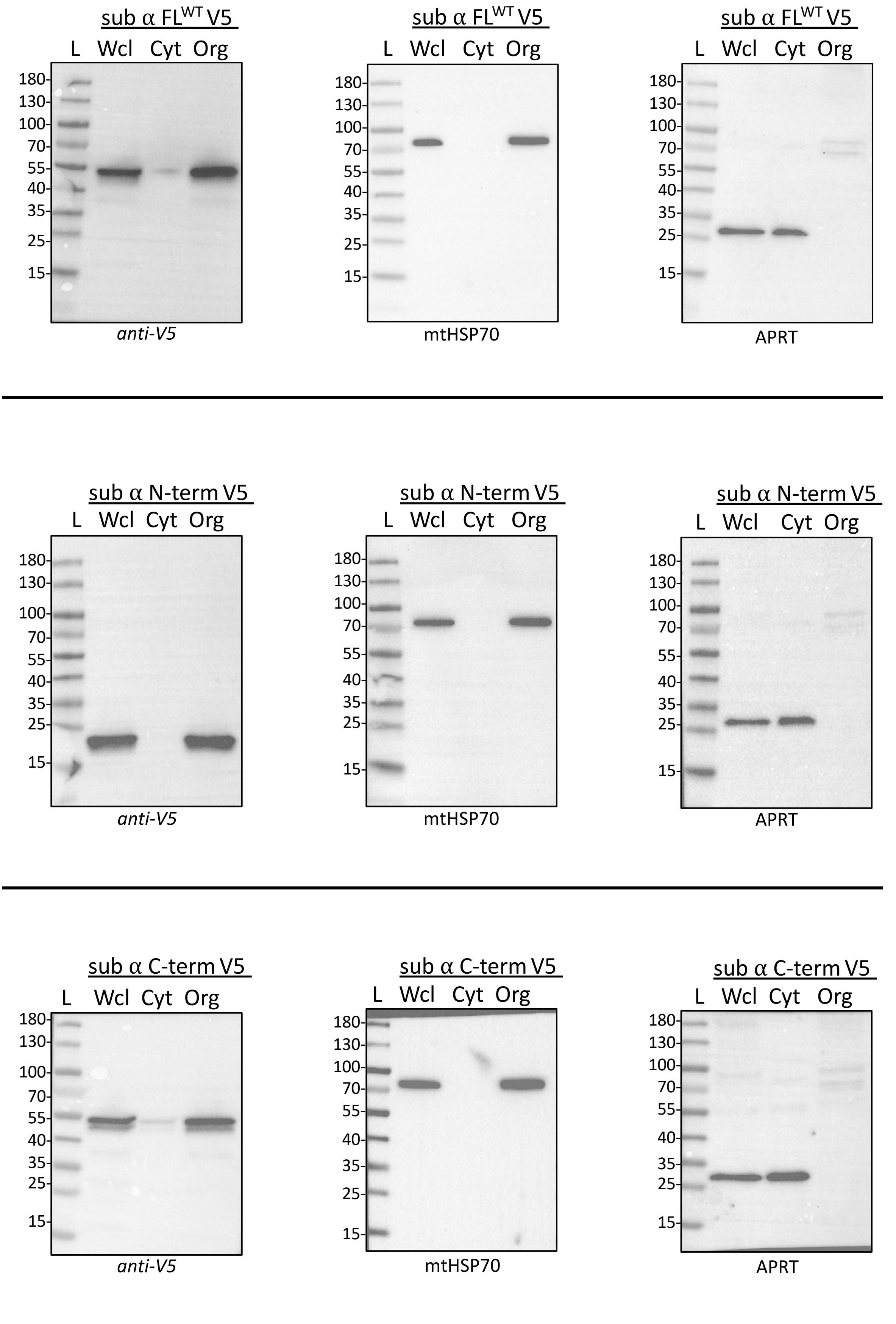
sub α FL^WT V5
L Wcl Cyt Org
180 130 100 70 55 40 35 25 15
anti-V5
mtHSP70
APRT
sub α N-term V5
L Wcl Cyt Org
180 130 100 70 55 40 35 25 15
anti-V5
mtHSP70
APRT
sub α C-term V5
L Wcl Cyt Org
180 130 100 70 55 40 35 25 15
anti-V5
mtHSP70
APRT

Figure S1C

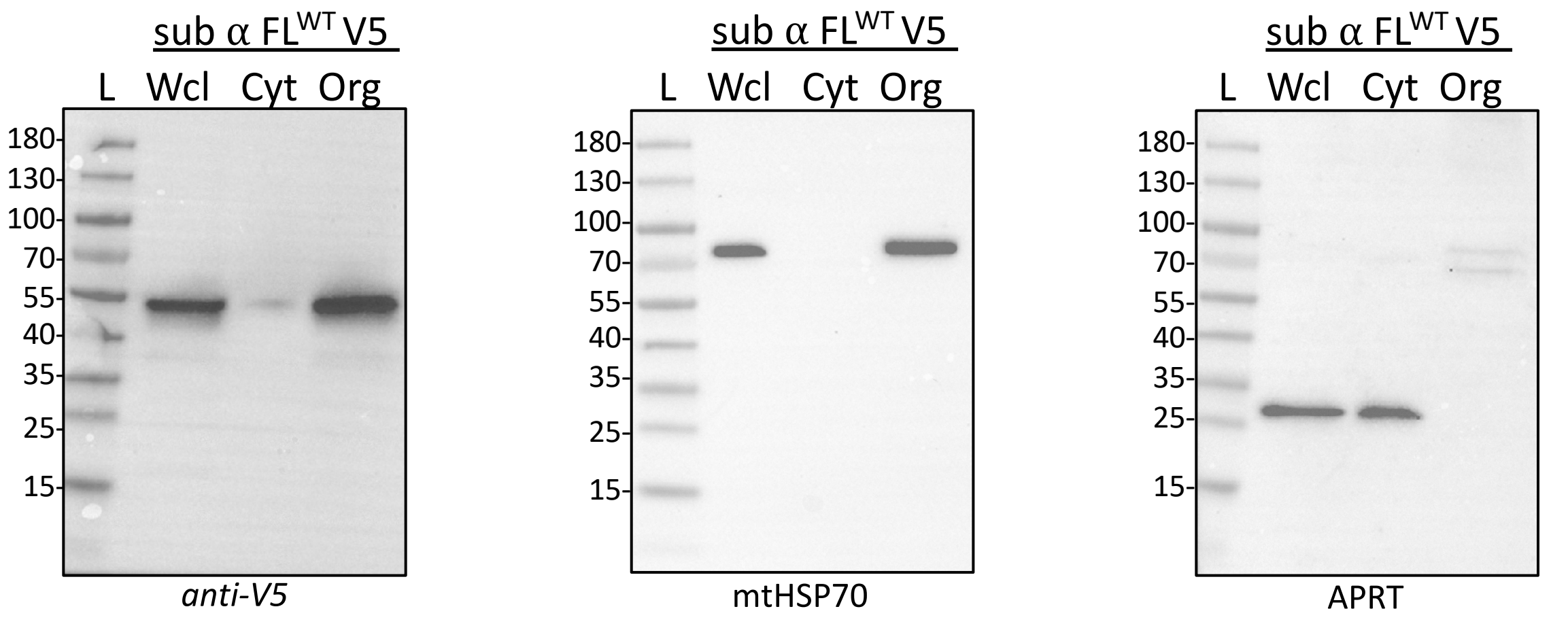

Figure S1D

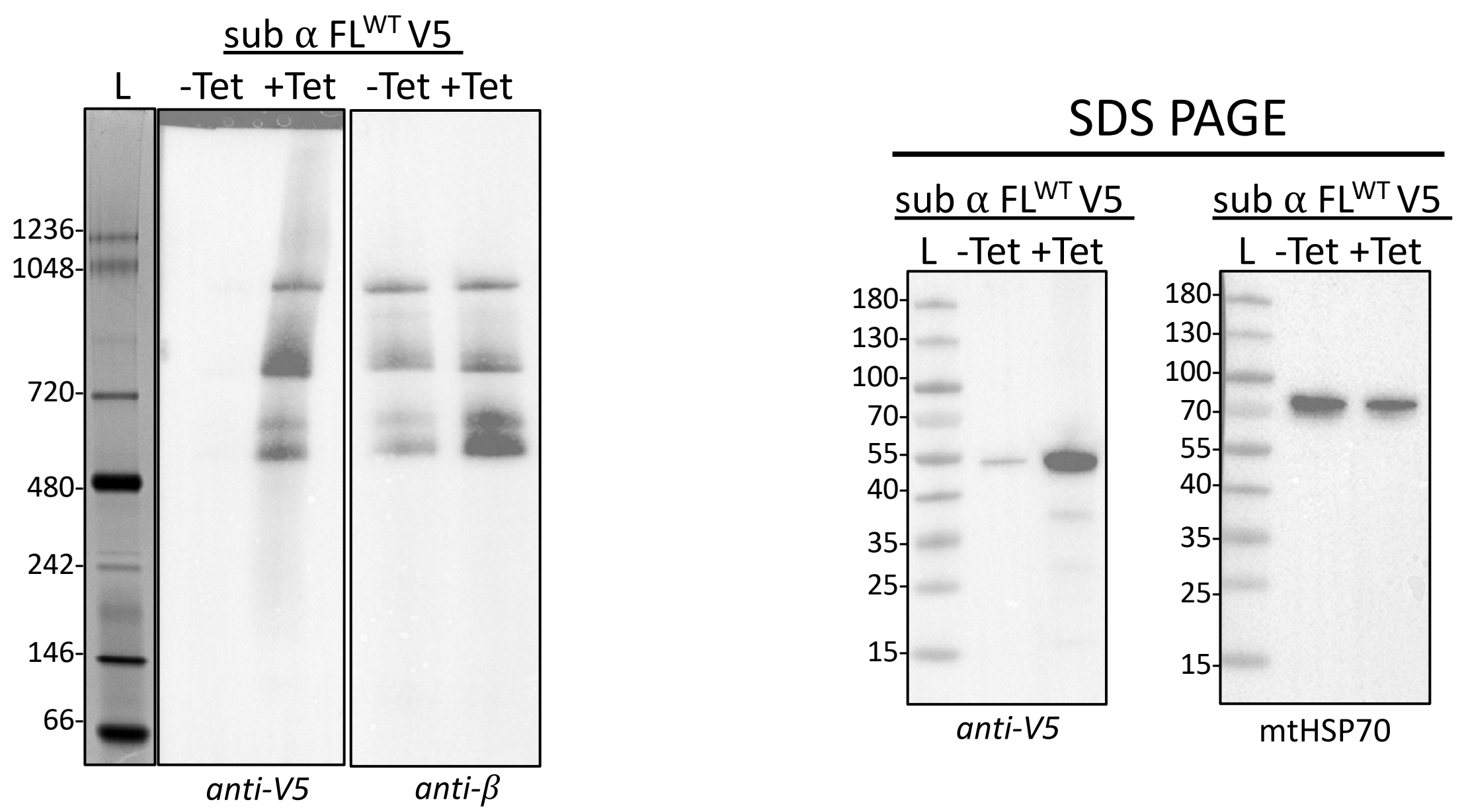

Figure S2 (1)

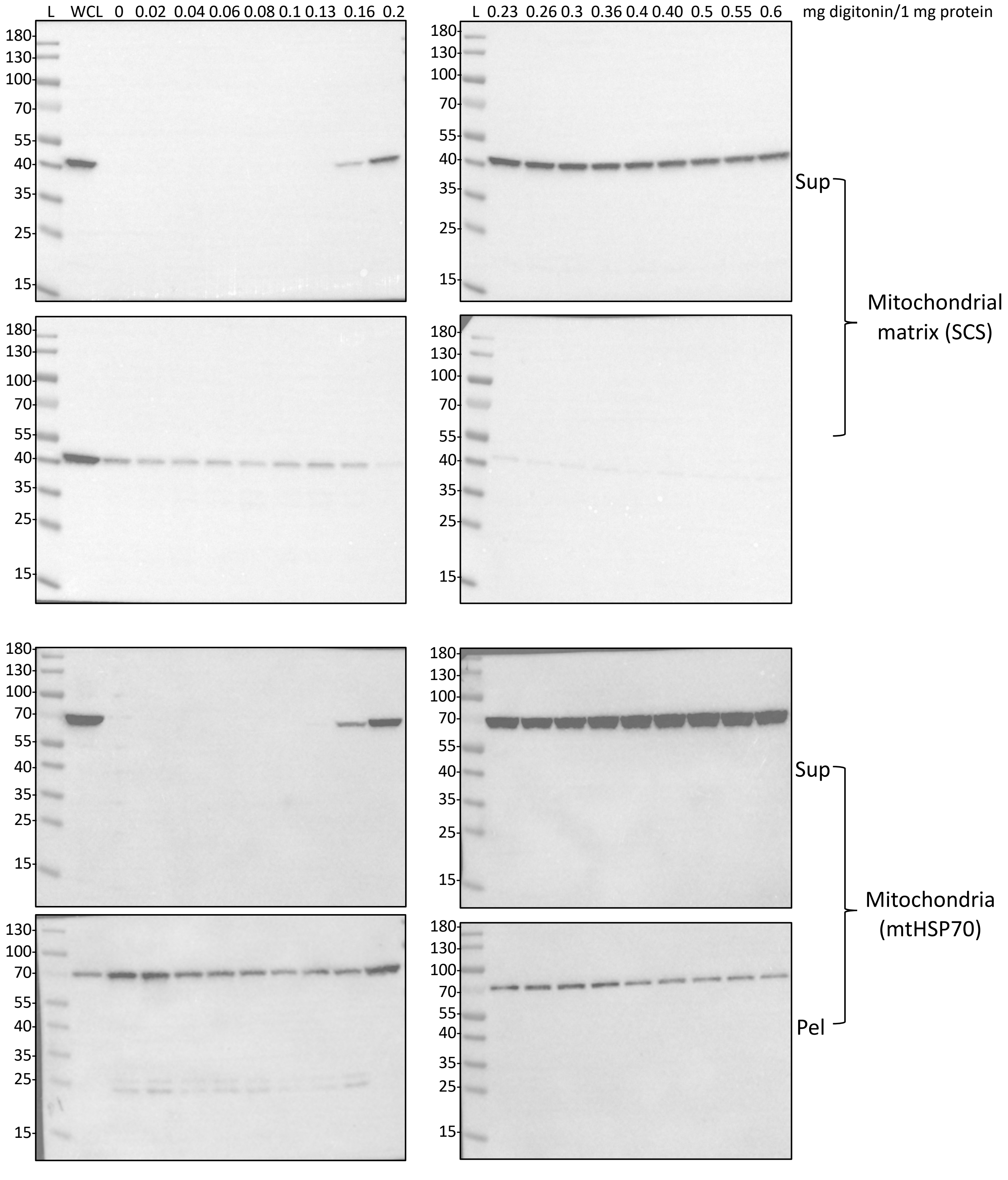

L WCL 0 0.02 0.04 0.06 0.08 0.1 0.13 0.16 0.2
L 0.23 0.26 0.3 0.36 0.4 0.40 0.5 0.55 0.6 mg digitonin/1 mg protein
180 130 100 70 55 40 35 25 15
Sup
Mitochondrial matrix (SCS)
Sup
Mitochondria (mtHSP70)
Pel

Figure S2 (2)

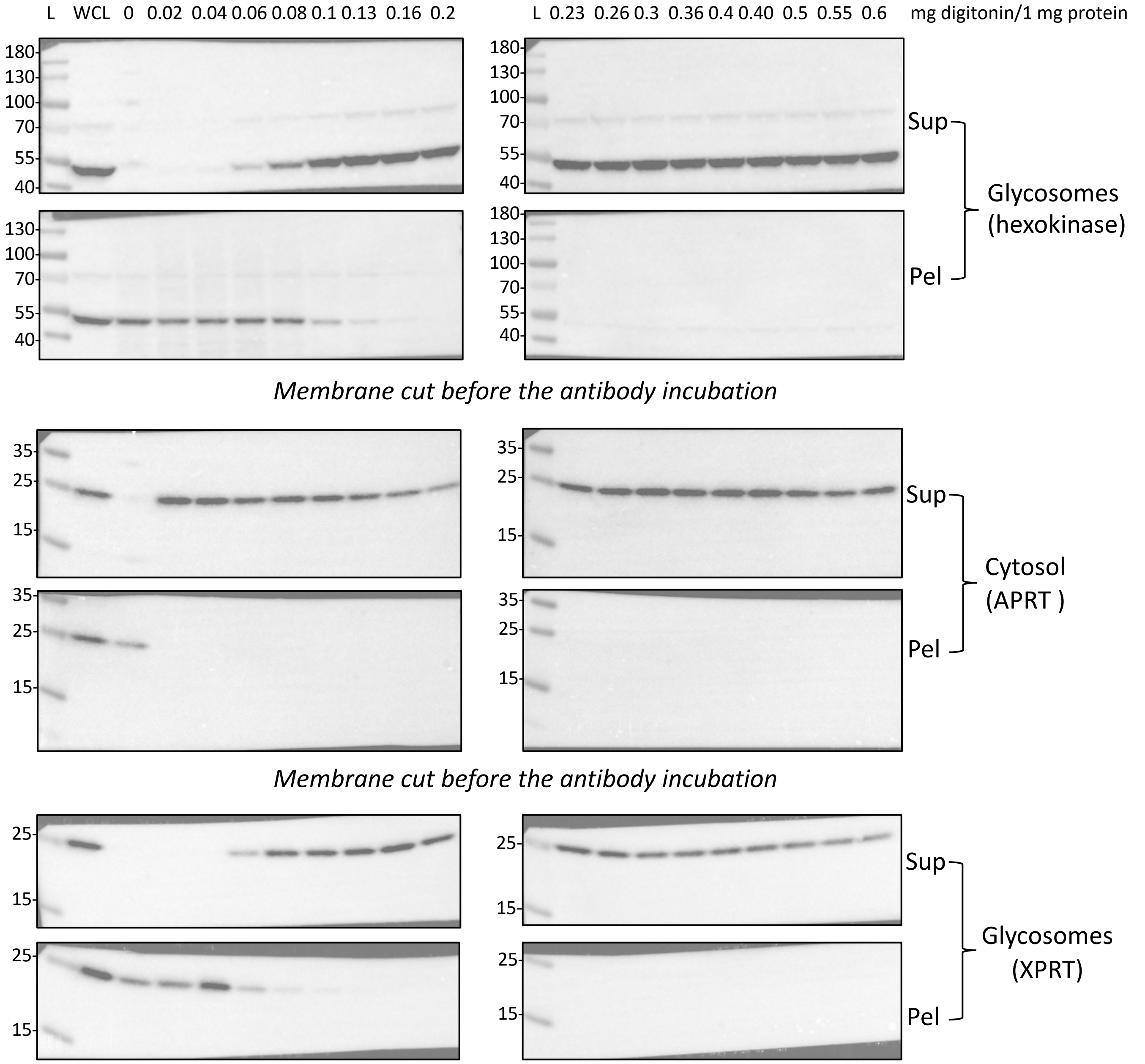
L WCL 0 0.02 0.04 0.06 0.08 0.1 0.13 0.16 0.2
L 0.23 0.26 0.3 0.36 0.4 0.40 0.5 0.55 0.6
mg digitonin/1 mg protein
180
130
100
70
55
40
Sup
Pel
Glycosomes (hexokinase)
Membrane cut before the antibody incubation
35
25
15
Cytosol (APRT)
Membrane cut before the antibody incubation
Glycosomes (XPRT)
