# Supplementary material for: Proteolytic processing of the unique F1-ATPase subunit α in procyclic Trypanosoma brucei
Source: Sci Rep. 2026 Apr 28;16:27787. doi: 10.1038/s41598-026-49778-4 (PMC13545225; doi:10.1038/s41598-026-49778-4)
Supplement: Supplementary file 2 [file 41598_2026_49778_MOESM3_ESM.pdf]

Figure S1

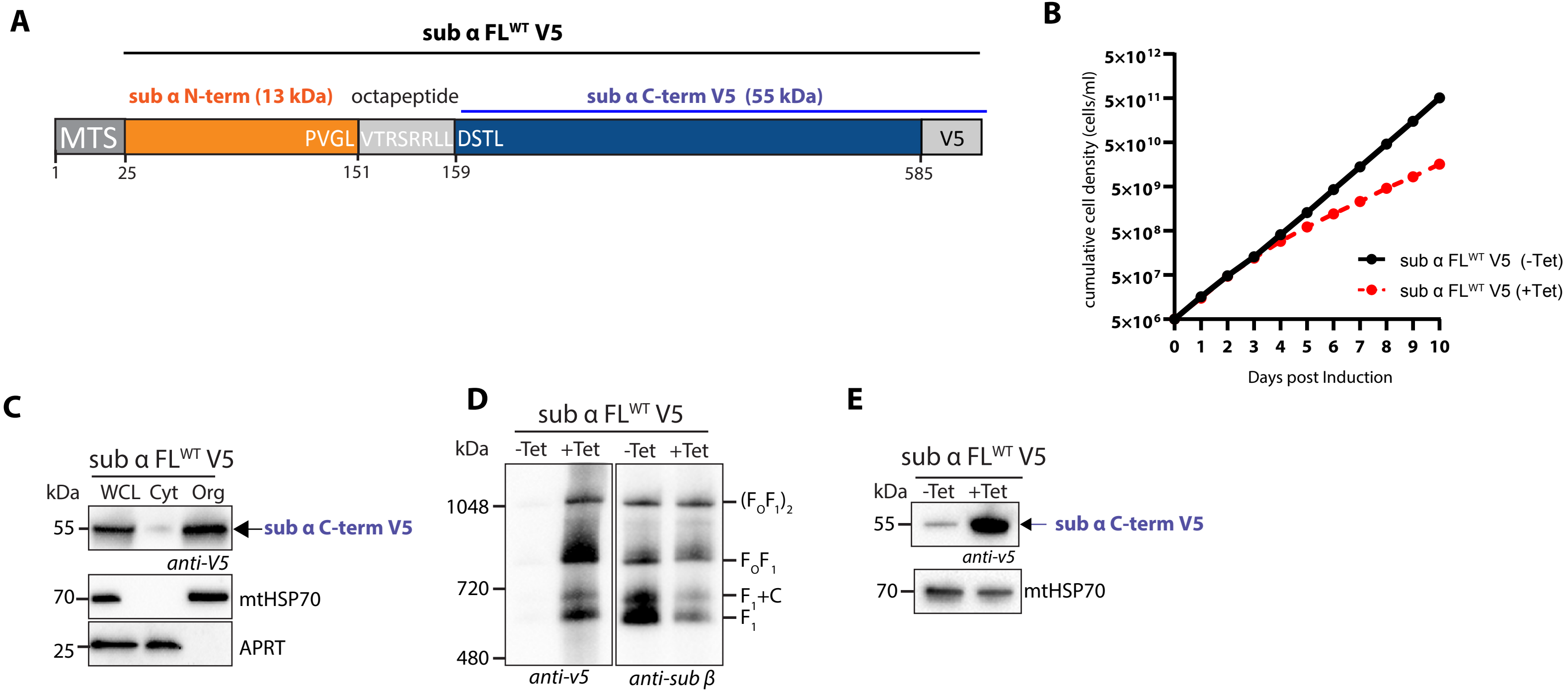

A
sub α FL^{WT} V5
sub α N-term (13 kDa)
octapeptide
sub α C-term V5 (55 kDa)
MTS
PVGL
VTRSRRLL
DSTL
V5
1
25
151
159
585
B
cumulative cell density (cells/ml)
5×10^{12}
5×10^{11}
5×10^{10}
5×10^{9}
5×10^{8}
5×10^{7}
5×10^{6}
0 1 2 3 4 5 6 7 8 9 10
Days post Induction
sub α FL^{WT} V5 (-Tet)
sub α FL^{WT} V5 (+Tet)
C
sub α FL^{WT} V5
kDa
WCL Cyt Org
55
70
25
sub α C-term V5
anti-V5
mtHSP70
APRT
D
sub α FL^{WT} V5
kDa
-Tet +Tet -Tet +Tet
1048
720
480
$(F_OF_1)_2$
F_OF_1
F_1+C
F_1
anti-v5
anti-sub β
E
sub α FL^{WT} V5
kDa
-Tet +Tet
55
70
sub α C-term V5
anti-v5
mtHSP70
