# Supplementary material for: Proteolytic processing of the unique F1-ATPase subunit α in procyclic Trypanosoma brucei
Source: Sci Rep. 2026 Apr 28;16:27787. doi: 10.1038/s41598-026-49778-4 (PMC13545225; doi:10.1038/s41598-026-49778-4)
Supplement: Supplementary file 3 [file 41598_2026_49778_MOESM4_ESM.pdf]

Figure S2

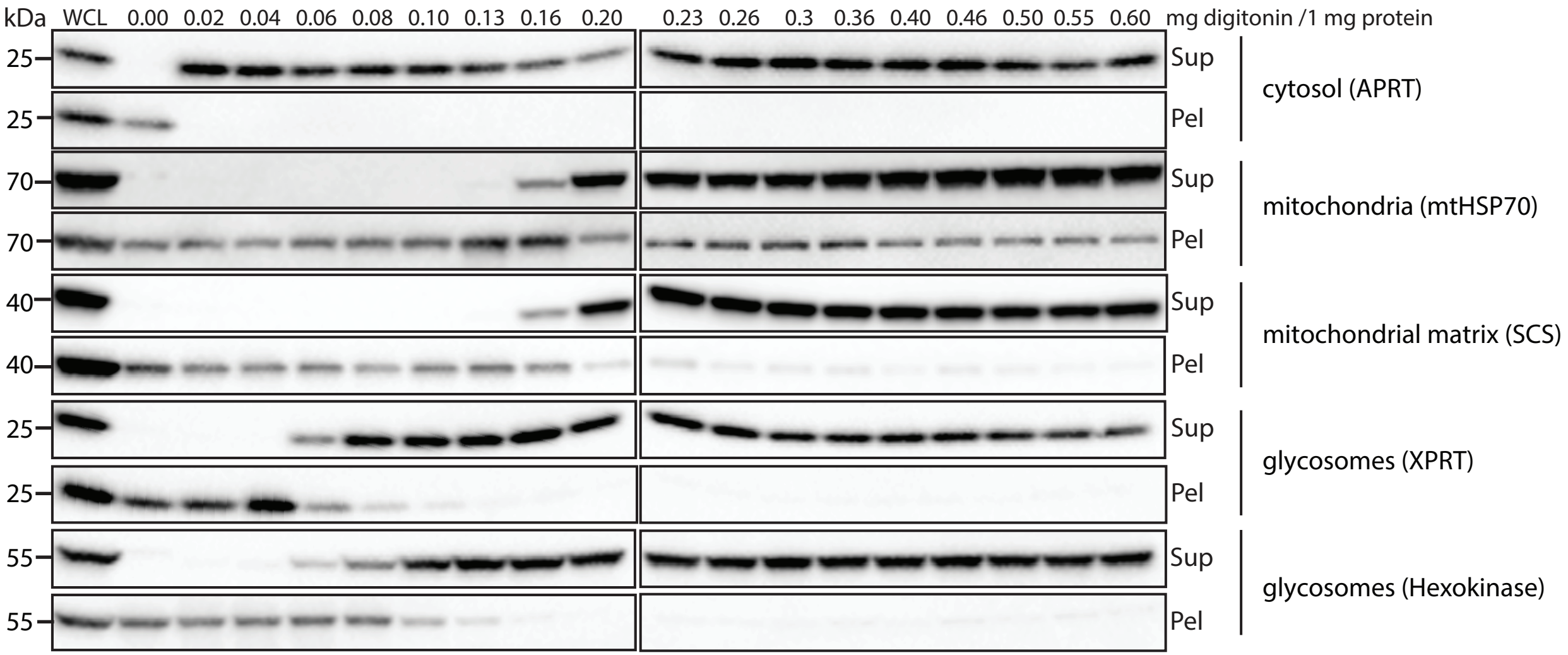
kDa
WCL
0.00
0.02
0.04
0.06
0.08
0.10
0.13
0.16
0.20
0.23
0.26
0.3
0.36
0.40
0.46
0.50
0.55
0.60
mg digitonin /1 mg protein
25
25
70
70
40
40
25
25
55
55
Sup
Pel
Sup
Pel
Sup
Pel
Sup
Pel
Sup
Pel
cytosol (APRT)
mitochondria (mtHSP70)
mitochondrial matrix (SCS)
glycosomes (XPRT)
glycosomes (Hexokinase)
