# Supplementary material for: Proteolytic processing of the unique F1-ATPase subunit α in procyclic Trypanosoma brucei
Source: Sci Rep. 2026 Apr 28;16:27787. doi: 10.1038/s41598-026-49778-4 (PMC13545225; doi:10.1038/s41598-026-49778-4)
Supplement: Supplementary file 4 [file 41598_2026_49778_MOESM5_ESM.docx]

**Supplemental Figure S1. Inducible expression of subunit α FL^WT^ V5 causes a growth reduction in *T. brucei.* A.** Schematic of *T. brucei* subunit α FL^WT^ with a C-terminal V5 tag. **B**. Growth curves of noninduced (-Tet) and induced (+Tet) subunit α FL^WT^ V5 cell lines grown in SDM-80 medium. **C**. Western blot analysis of whole cell lysate (WCL), cytosol (Cyt) and digitonin-extracted enriched organellar fractions (Org) from *T. brucei* expressing subunit α FL^WT^ tagged with a V5 epitope. The relevant sizes of the protein marker are indicated on the left. Antibodies recognizing mitochondrial and cytosolic markers, mtHSP70 and APRT, respectively, were used as controls for proper fractionation. **D.** Representative immunoblots of mitochondrial lysates from *T. brucei* subunit α FL^WT^ V5 cell lines either noninduced (-Tet) or induced for 2 days (+Tet) were resolved by BN-PAGE and probed with antibodies against the V5 epitope and F_1_-ATPase subunit β. Positions of high molecular weight marker are shown. **E.** Mitochondrial lysates from **D** were separated on SDS PAGE and probed with antibodies against V5. Immunoblot with anti-mtHSP70 antibody served as a loading control.

**Supplemental Figure S2. Digitonin latency of *T. brucei* PF427 cells.** Immunoblots of whole cell lysates (WCL) and subcellular fractions obtained from treatments with digitonin at increasing concentrations. Antibodies recognizing specific proteins localized in either the cytosol, mitochondrion, mitochondrial matrix or glycosomes were applied. Protein marker sizes are indicated on the left.

**Supplemental Table S1. *T. brucei* transgenic cell lines generated in this study.**

**Supplemental Table S2. List of oligonucleotides used in this study.**

**Supplemental Table S3. List of used antibodies in this study.**

**Supplemental Table S4. Raw data supporting Figure 4E and 4F analysis.**
